# *Morphogenia*: a new genus of the Neotropical tribe Jubini (Coleoptera, Staphylinidae, Pselaphinae) from the Brazilian Amazon

**DOI:** 10.3897/zookeys.373.6788

**Published:** 2014-01-23

**Authors:** Joseph Parker

**Affiliations:** 1 Department of Genetics and Development, Columbia University, 701 West 168th Street, New York, NY 10032, USA; 2 Division of Invertebrate Zoology, American Museum of Natural History, New York, NY 10024, USA

**Keywords:** Pselaphinae, Jubini, Neotropical, Brazil, new genus, new species

## Abstract

A new genus and species of the large Neotropical pselaphine tribe Jubini is described from Manaus, Brazil, based on material preserved in the Natural History Museum, London. *Morphogenia struhli* **gen.** et **sp. n.** represents the possible sister taxon of the abundant and speciose genus *Barrojuba* Park, sharing with it the putatively derived condition of anterolaterally shifted vertexal foveae, producing a smoothly convex vertex devoid of fovea or sulci. However, unlike *Barrojuba*, *Morphogenia* retains a plesiomorphic antebasal sulcus on the pronotum in both sexes, and additionally lacks elaborate abdominal fovea-like pockets and teeth on the lateral margins of the pronotum that are typical of *Barrojuba*. The genus is also unusual among jubine genera in lacking the characteristic V- or Y-shaped gular carina. In contrast to the commonly-collected *Barrojuba*, specimens of *Morphogenia* are absent in extensive jubine collections housed in museums in the United States, indicating that the new taxon may be relatively scarce or localised.

## Introduction

The pselaphine tribe Jubini is an entirely Neotropical radiation and a major component of the South American rove beetle fauna (Park 1942, 1952). The tribe is highly distinctive, with a wide mentum and maxillary cardos that project anteriorly, to the point that they are sometimes visible in dorsal view. A conspicuous V- or Y-shaped gular carina commonly adorns the underside of the triangular head, and the pronotum is also of a characteristic form, often abruptly constricted at the base.

Thirteen genera of Jubini have been described (Newton and Chandler 1989, Chandler 1999), with several, including *Jubus* Schaufuss, *Sebaga* Raffray and *Barrojuba* Park being abundant and potentially very species rich elements of the Neotropical forest floor. Despite their ubiquity, and the accumulation of a mass of new material from collecting efforts over the past several decades, little work has been conducted on Jubini since the creation of the majority of genera over a century ago. One exception is the genus *Barrojuba*, erected by Park (1942) and subsequently revised by (Chandler 1983, 1988), and now including fourteen species. *Barrojuba* is distinctive in possessing an overtly simplified and smoothly convex vertex, resulting from an evolutionary loss of sulci and an anterolateral repositioning of the vertexal foveae (the dorsal tentorial pits) to the vicinity of the postantennal notches.

Investigation of the pselaphine collection at the Natural History Museum, London (NHM), revealed undescribed jubine material collected during the 1990s from the Smithsonian Tropical Research Institute/INPA Biological Dynamics of Forest Fragments Project in Manaus, Brazil. Among this material were specimens with apparent affinities to *Barrojuba*, but differing in crucial aspects that warrant their placement in a new genus. Here, the new genus is described and illustrated, its sexual dimorphism documented, and putative phylogenetic relationships discussed.

## Methods

For morphological description, specimens were removed from mounts and relaxed in a Sodium Dodecyl Sulphate-based buffer with Proteinase K, and then transferred into ethanol for observation with stereo microscopy, or into 1:1 ethanol : glycerol for imaging with a Zeiss compound microscope. Genitalia and terminal segments were dissected and mounted in temporary glycerol slide mounts, and are stored in glycerol microvials pinned with their respective specimens. Photographs of specimens were taken by using a Visionary Digital photomicrographic apparatus with Infinity optics and a Canon 60D camera, installed at the American Museum of Natural History, New York. Montage images were constructed from stacks using Helicon Focus. DNA from two paratype females was extracted according to a non-destructive protocol outlined previously (Parker and Maruyama 2013). The symbol “//” is used to separate different data labels attached to the specimens. The terminology used to describe the foveal system follows Park (1942), as modified by Chandler (2001), except that the terms “mesoventral” and “metaventral” are used in place of “mesosternal” and “metasternal”, following the discussion of Herman (2013).

## Taxonomy

### Morphogenia gen. n.

http://zoobank.org/4368E5BC-73B0-443D-AE2C-020F58EFD36C

http://species-id.net/wiki/Morphogenia

#### Type species.

*Morphogenia struhli*, here designated.

#### Diagnosis.

*Morphogenia*, and its only species, *Morphogenia struhli*, can be distinguished from all other known jubine genera by the following combination of characters: (1) smoothly convex vertex lacking sulci, and afoveate due to anterolateral shift of vertexal foveae into vicinity of postantennal notches anterior to eyes; (2) absence of V- or Y-shaped gular carina; (3) pronotum with margins smooth, lacking lateral spines, and with a simple, well-defined transverse antebasal sulcus in both sexes; (4) abdomen lacking fovea-like cuticular pockets at bases of tergites V–VII and sternites V–VII (but tergite IV and sternite IV with true mediobasal foveae present).

#### Description.

**Figures 1–4.**
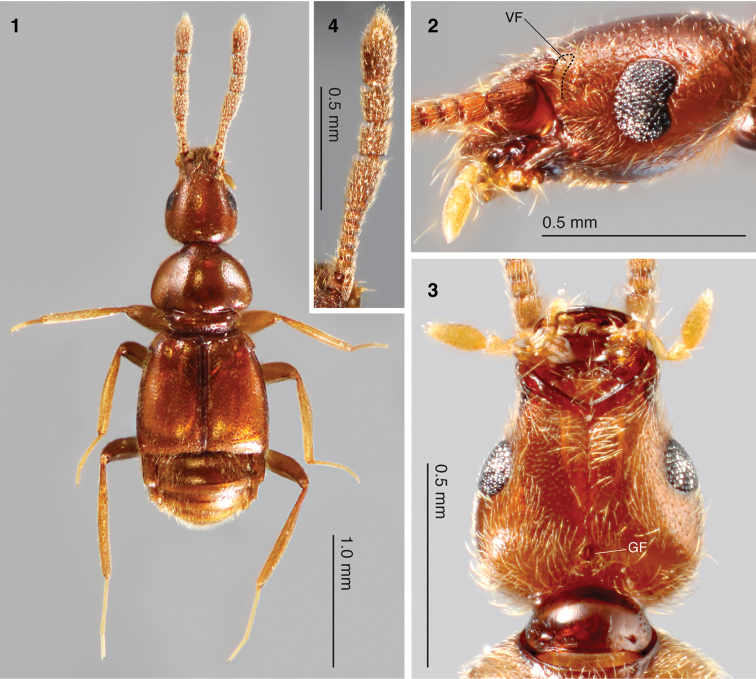
*Morphogenia struhli* sp. n. holotype male. **1** dorsal habitus **2** head, lateral view, with position of vertexal fovea (VF) indicated at dorsal extreme of postantennal notch (region enclosed by dashed line) **3** head venter showing gular fovea (GF). Note the absence of a gular carina **4** right antenna, dorsal view.

Body length ~3 mm (Fig. 1). Form relatively flattened and broadened posteriorly, with compact abdomen and elongate legs.

**Figures 5–15.**
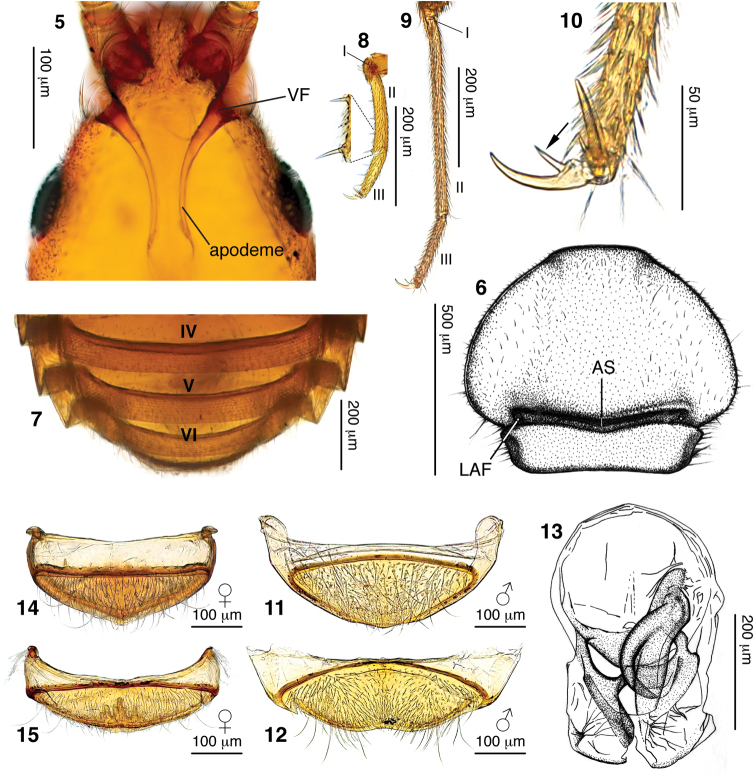
Diagnostic characters of *Morphogenia struhli* sp. n. **5** compound micrograph of male head showing anterolaterally shifted vertexal fovea (VF) and apodemes of the tentorium **6** male pronotum with antebasal sulcus (AS) and lateral antebasal fovea (LAF) indicated **7** male dorsal abdominal segments showing absence of fovea-like cuticular pockets at the bases of tergites V–VII **8** male left protarsus with tarsomeres indicated. A magnified portion of the ventral face of tarsomere II shows the large spines of possible raptorial function **9** male left metatarsus with tarsomeres indicated. Note that tarsi in **8** and **9** are to the same scale. **10** male left hind tarsal claw, with arrow indicating internal spike **11** male tergite VIII **12** male sternite VIII **13** dorsal view of aedeagus **14** female tergite VIII **15** female sternite VIII. In **11–15** all structures are orientated with anterior to the top.

**Head:** Approximately triangular (Figs 1, 18), 1.3× wider than long; without distinct frontal rostrum and lacking prominent, raised antennal tubercles. Vertex smoothly convex, devoid of foveae or sulci, lateral margins incised behind antennal sockets by postantennal notches (region enclosed by dashed line in Fig. 2). Antennae separated by 1/3 maximum head width. Foveae of apparent homology to the vertexal foveae of other Pselaphinae shifted from vertex, situated instead on frontolateral margins, recessed into the top of postantennal notches (Figs 2, 5). Apodemes of tentorium extending from these foveae (Fig. 5) and converging on gular foveae with single opening (Fig. 3). Lateral margins of head smoothly rounded dorsoventrally, lacking ocular mandibular carina. Venter lacking any trace of gular carina (Fig. 3; a medial sutural line can be weakly detected). Antennae (Fig. 4) with 11 antennomeres, with club formed by enlarged antennomeres VIII–XI. Maxillary palpi comprised of five palpomeres, with small triangular palpomeres III and fusiform palpomeres IV (Fig. 3). Maxillary cardos projecting anteriorly, reaching slightly beyond sides of mandibles, with single long setae positioned at apex.

**Figures 16–19.**
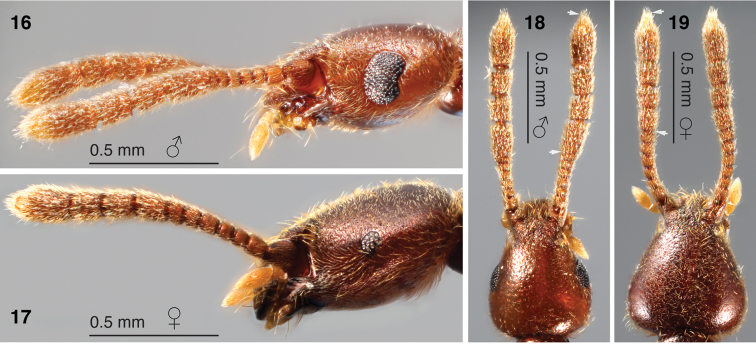
Sexual dimorphism in head morphology **16** male head, lateral view **17** female head, lateral view **18** male head dorsal view **19** female head dorsal view. Arrows in **18** and **19** indicate extent of the antennal club.

**Thorax:** Pronotum (Fig. 6) moderately transverse, 1.4× wider than head and similar in length, obcordate in shape, approximately semicircular before abrupt constriction in basal quarter. Lateral margins before constriction smoothly rounded, without spines or teeth. Pronotal disc simple and convex, lacking foveae or sulci. Typical, deep and well-impressed antebasal sulcus present, demarcating point of pronotal constriction. Lateral antebasal foveae present, median antebasal fovea absent. Prosternum with lateral procoxal foveae. Mesoventrite with single unpaired median mesoventral fovea, lateral mesoventral foveae and lateral mesocoxal foveae. Metaventrite with lateral metaventral foveae, with median carina from 1/3 segment length to posterior margin.

**Abdomen:** Abdomen 2/3 length of elytra (measured along suture). Five tergites (IV–VIII) evident. Tergites with broad, angularly-projecting paratergites on segments III–VI and smaller paratergites on VII (Fig. 7). Tergite IV longest, 2.7× tergite V length, with posterior tergites becoming shorter and narrower. Tergite IV with mediobasal foveae present in basal sulcus. Six sternites (III–VIII) evident; penial plate apparently internalized, not externally visible, with genital aperture formed by contiguous apical margins of tergite and sternite VIII (Figs 11, 12). Apical margin of sternite III entire, uninterrupted by metacoxae. Sternite IV longest, mediobasal foveae present. All tergites and sternites lacking fovea-like cuticular pockets at their bases (Fig. 7 shows bases of tergites V–VII revealed in cleared specimen).

**Elytra:** 1.3× longer than pronotum, broadening gradually until narrowing just before apices. With sinuate transverse basal carina; sutural foveae and single median basal foveae present but largely obscured by arcing of basal carina. Humeri indented by impressed bases of humeral sulci; humeral sulci extending length of elytra, with humeral foveae at base. Sutural striae entire.

**Legs:** All pairs of coxae contiguous. Coxae all carinate along length of external face. Procoxal length greater than half femoral length, procoxae strongly conically projecting ventrally. Mesocoxae shorter than procoxae, moderately conically projecting, orientated somewhat posteriorly. Metacoxae transverse-conical, spanning from ventral midline to metaventral margin, and projecting posteriorly. All trochanters short, with negligible separation of coxal apex and femoral base (“brachysceline” type). Femora and tibiae simple, lacking modifications. Profemora somewhat thickened. Tarsi (Figs 8, 9) 3-segmented with short tarsomeres I; tarsomeres II longest. Metatarsi especially elongate. Tarsi with two claws of equal size.

#### Etymology.

Morphogens are gradient-forming molecules that specify positional information and govern tissue growth during animal development. The generic name acknowledges the pervasive role of morphogens in sculpting organismal morphology. The gender is feminine.

### Morphogenia struhli sp. n.

http://zoobank.org/21B35D69-23FB-42AE-8601-80AEFEB986CE

http://species-id.net/wiki/Morphogenia_struhli

#### Type material.

Holotype ♂: “88 1 // Leaf litter, Winkler method. Terra firmé fst. // **BRAZIL:** Manaus, A.M. INPA/Smithsonian Res. 2°25'S, 59°50'W, R. Didham, i. 1994 // BMNH{E} 2003-84.”. Paratypes (2 ♀♀): “89 7 // Leaf litter, Winkler method. Terra firmé fst. // **BRAZIL:** Manaus, A.M. INPA/Smithsonian Res. 2°25'S, 59°50'W, R. Didham, i. 1994//BMNH{E} 2003-84 // (pink label) 0749”. “68 12 // Leaf litter, Winkler method. Terra firmé fst. // **BRAZIL:** Manaus, A.M. INPA/Smithsonian Res. 2°25'S, 59°50'W, R. Didham, i. 1994 // BMNH{E} 2003-84”. All material is deposited in the Coleoptera collection of the Department of Entomology, Natural History Museum, London.

#### Description.

Body length 2.9 mm (Fig. 1). Holotype male somewhat teneral; body colour of female paratypes dark reddish-purple (e.g. Figs 17, 19) with appendages lighter in colour. Dorsal regions shiny, with shallow punctures and sparse setae in most areas.

**Head:** Length 0.71 mm from occipital constriction to clypeus; width across widest point (posterior to eyes) 0.55 mm. Margins broadly and smoothly rounded from eyes to base (Figs 1, 18). Margins narrowing anterior to eyes before broadening slightly to clypeus. Setae sparse on vertex and becoming longer and denser towards clypeus. Anterolaterally-shifted vertexal fovea concealed in postantennal notches (region indicated in Fig. 2) by several long, apically-directed setae (Fig. 5). Eyes (Fig. 2) large with ~105 ommatidia, broadly crescent-shaped with shallow ocular canthi at posterior margin. Antennae received by triangular frontolateral excavations with carinate edges (Fig. 2). Antennal (Fig. 4) length 1.18 mm, with scape weakly transverse in dorsal view, pedicel slightly narrower and subquadrate. Antennomeres III–V subequal in width, slightly narrower and shorter than pedical, with segments becoming progressively longer apically; VI and VII transverse and subequal in length; VII wider than VI and approaching width of segment VIII. Antennomeres VIII–X with carinate apical and basal margins, roughly obconical and equal in width. Antennomere VIII slightly longer than preceding three segments, 2/3 longer than wide; antennomeres IX and X 0.7× length of VIII; XI longest, 1.8× length of VIII, with carinate basal margin, widest in apical half before tapering to apex. Antennomeres densely covered with apically-directed setae. Apical pseudosegment of maxillary palpus continuous with external face of palpomere IV and pointing slightly mesially.

**Thorax:** Pronotum (Fig. 6) length 0.68 mm, width 0.77 mm at widest point. Disc region sparsely setiferous. Lateral margins behind antebasal sulcus with several long, laterally-projecting setae. Prosternum with medial region in front of procoxae with long setae covering setiferous lateral procoxal fovea. Episternal areas with moderately dense, shorter setae orientated somewhat dorsally. Short lateral prosternal carinae extending briefly from sides of procoxal cavities. Mesoventral plate moderately setiferous, meso-epiventral regions largely glabrous. Metasternum with sparse, dorsoapically-pointing aciculate setae.

**Abdomen:** Dorsal abdominal length (with segments contracted) 0.57 mm. Tergite lengths: IV= 0.32 mm, V = 0.12 mm, VI = 0.10 mm. Apical margin of sternite IV with male-specific medially positioned small, round tubercle covered in small setae. Male tergite VIII (Fig. 11) with basal margin of sclerite weakly convex; with uniform moderately dense distribution of long setae. Sternite VIII (Fig. 12) with basal margin of sclerite strongly convex; mediobasal region with sparse, short setae becoming much denser towards apical margin; lateral areas of sternite with much longer setae of moderate density. Apical margin of sternite VIII medially depressed to receive corresponding apex of tergite VIII.

**Elytra and flight wings:** Elytra with scattered, short setae. Elytral length along suture 0.87 mm; width at widest point 0.5 mm. Full flight wings present.

**Legs:** Femora brownish-red, slightly lighter than body; tibiae and tarsi lighter than femora, yellowish (Fig. 1). Tarsi of increasing relative length: protarsi 0.5× protibial length; mesotarsi 0.6× protibial length; metatarsi 0.7× metatibial length. Protarsomeres II 1.7× tarsomere III length (Fig. 8). Mesotarsomeres II 1.8× tarsomere III length. Metatarsomeres II especially long (Fig. 9), 2.3× tarsomere III length. All tarsal claws with inner spine present on ventral face (Fig. 10). Pro- and mesotarsi with additional spines on ventral side of tarsomeres II and III (Fig. 8).

**Aedeagus:** Length 0.43 mm, width 0.27 mm at widest point (Fig. 13). Asymmetric, dorsoventrally relatively flattened, with large spherical and very weakly sclerotized basal bulb. Lacking obvious remnants of parameres. Complex, more heavily sclerotized medioapical piece with “hooked” apophysis extending apically from right side of basal bulb.

#### Sexual dimorphism.

Female dimensions similar to male. Males with large crescent shaped eyes (Fig. 16); female eyes very small and approximately oval, consisting of ~12 ommatidia (Fig. 17). Vertex of female head (Fig. 19) broader at base than that of male (Fig. 18). Antennal club (VIII–XI) twice as long as preceding segments combined in male (Fig. 18), only 1/3 as long as preceding segments combined in female (Fig. 19). Medial sexual patch on sternite IV absent in female, and flight wings lacking. Female tergite VIII (Fig. 14) with basal margin of sclerites shallowly concave; with moderate density of long setae and two pairs of much longer setae. Female sternite VIII (Fig. 15) with basal margin of sclerite weakly convex; covered in moderate density of short setae and several pairs of much longer setae. Apex with internally-projecting cuticular protuberance.

#### Etymology.

The type species of *Morphogenia* is named in honour of Dr. Gary Struhl, developmental biologist, whose *Drosophila* studies have yielded great insights into the genetic control of animal development.

#### Biology.

*Morphogenia* was collected from rainforest litter—the typical habitat of jubines. Like other pselaphines, jubines are most likely predators of soil microarthropods. The spines on the ventral face of the pro- and mesotarsi of *Morphogenia struhli* (Fig. 8) may serve a raptorial function. Prior to photography, a filament protruding from the mouthparts of one female paratype was removed. A subsequent survey of jubine specimens in a range of genera have revealed similar filaments, which appear to be dried, threadlike residues of glutinous secretions from the enlarged maxillary cardos that are characteristic of the tribe. Such structures suggest the evolution of a novel mode of feeding or prey capture in Jubini (J. Parker & C. Carlton unpublished observations).

## Discussion

### Phylogenetic relationships of *Morphogenia*

Creation of a new genus, *Morphogenia*, is supported by cladistic interpretation of the suit of adult character states presented by the NHM material. Among jubines, *Morphogenia* appears to be allied to *Barrojuba* based on its putatively derived dorsal head morphology: the lack of sulci, and the proposed synapomorphy of anterolaterally shifted vertexal foveae that sit in the post-antennal regions anterior to the eyes. A smoothly convex vertex is present in *Arctophysis* Reitter, another jubine genus, but here the vertexal foveae have been evolutionarily lost rather than repositioned, and vestiges of the apodemes of the tentorium remain connected to the medial vertex (visible in cleared specimens). The dorsal head morphology of *Barrojuba* and *Morphogenia* is perhaps most closely approached by *Endytocera* Sharp, where the vertexal foveae sit somewhat anterolaterally on the head vertex, but are recessed into deep S-shaped sulci that extend from the gular region dorsally between postantennal notch and eye, merging apically at the interantennal area of the frons.

Despite their similar vertexal morphologies, *Morphogenia* and *Barrojuba* differ markedly in several important characters. *Barrojuba* lacks a typical (straight and simple) antebasal sulcus on the pronotum in the male sex. Instead, this part of the pronotum is variously modified and impressed (females of a few species have what approaches a typical sulcus; D.S. Chandler pers. comm.). In contrast, *Morphogenia* retains the plesiomorphic condition of a conventional antebasal sulcus in both sexes. A further autapomorphy of *Barrojuba* is the presence of large, fovea-like cuticular invaginations at the bases of tergites V–VII and sternites V–VIII. Such invaginations are absent in *Morphogenia*. Hence, *Morphogenia* possesses only one of three putatively derived character states of *Barrojuba*, arguing for its distinctiveness and proposed sister taxon status.

A large number of other characters of systematic utility separate *Barrojuba* and *Morphogenia*, in combination supporting the reciprocal monophyly of the two genera. However, for several such characters, whether these differences represent gains or losses in the respective genus is presently unclear. *Barrojuba* has tooth-like acuminations at the lateral edges of the pronotum that are absent in *Morphogenia*, but seemingly homologous structures are present in many jubine genera, implying a possible loss in *Morphogenia*. Most strikingly, *Morphogenia* lacks any trace of a V- or Y-shaped gular carina—a character found in all jubine genera except *Balega* Reitter, *Pselaphomorphus* Motschulsky, and *Phamisus* Aubé. Such an absence may again be consistent with a loss occurring in *Morphogenia*. The tarsal claws of *Morphogenia* are equal in size, while in *Barrojuba* they are unequal. Equally-sized claws are plesiomorphic in pselaphines, but instances of evolutionary reversal from inequality may have occurred (Parker and Maruyama 2013), and this character state varies among jubine genera, and even within them in the case of *Jubus* Schaufuss, the largest genus of the tribe (Park 1942). A further discrepancy between the two genera exists in the pattern of sexual dimorphism. In *Barrojuba*, the male antennal club is comprised of elongate antennomeres VII–XI or VIII–XI, depending on the species. The antennomeres of the female club are relatively less elongate than those of the respective male, and the club contains one fewer antennomere. In *Morphogenia*, the female antennomeres are again relatively less elongate than those of the male, but the number of antennomeres forming the club is equal in both sexes (four).

Interpreting transitions in these more broadly distributed or sexually dimorphic characters will require a thorough phylogenetic analysis of the entire tribe. Extensive collections of Neotropical pselaphines, accumulated from expeditions spanning decades, are housed in the Field Museum of Natural History, American Museum of Natural History, Louisiana State Arthropod Museum and University of Kansas Biodiversity Institute. Screening these collections for *Morphogenia* yielded no further specimens, implying that the genus may be relatively scarce or geographically localised. Fortunately, despite the age of the three known specimens, which were collected almost twenty years ago, DNA extraction from the two female paratypes of *Morphogenia struhli* was successful. Partial gene sequences have been obtained that may help resolve the placement of the new genus in a future molecular phylogenetic analysis of Jubini.

## Supplementary Material

XML Treatment for Morphogenia

XML Treatment for Morphogenia struhli
